# Anatomical Study of Intrahemispheric Association Fibers in the Brains of Capuchin Monkeys (*Sapajus* sp.)

**DOI:** 10.1155/2015/648128

**Published:** 2015-11-29

**Authors:** Kellen Christina Malheiros Borges, Hisao Nishijo, Tales Alexandre Aversi-Ferreira, Jussara Rocha Ferreira, Leonardo Ferreira Caixeta

**Affiliations:** ^1^Department of Biology, Academic Areas, Federal Institute of Goiás, 75131-45 Anápolis, GO, Brazil; ^2^System Emotional Science, Graduate School of Medicine and Pharmaceutical Sciences, University of Toyama, Toyama 930-0194, Japan; ^3^Laboratory of Anthropology, Biochemistry, Neuroscience and Primate Behavior (LABINECOP), Federal University of Tocantins, 77001-090 Palmas, TO, Brazil; ^4^School of Medicine, University of Brasília, 70910-900 Brasília, DF, Brazil; ^5^Behavioral Neurology Unit, Hospital das Clínicas, Federal University of Goiás, 74605-020 Goiânia, GO, Brazil

## Abstract

Previous studies suggest that the complexity of fiber connections in the brain plays a key role in the evolutionary process of the primate brain and behaviors. The patterns of brain fiber systems have been studied in detail in many nonhuman primates, but not in* Sapajus* sp. Behavioral studies indicated that* Sapajus* sp. (bearded capuchins) show highly cognitive behaviors such as tool use comparable to those in other nonhuman primates. To compare the brain fiber systems in capuchins with those in other nonhuman primates and humans, the intrahemispheric fibers systems in 24 cerebral hemispheres of* Sapajus* were dissected by a freezing-thawing procedure. Dissection of the hemispheres in lateral view indicated short arcuate fibers, uncinate fasciculus, and inferior longitudinal fasciculus, while that in a medial view indicated short arcuate fibers, the cingulum united with the superior longitudinal fasciculus, and inferior longitudinal fasciculus. The results showed that the fiber systems in* Sapajus* are comparable to those in rhesus and humans, except for a lack of independent superior longitudinal fasciculus and cingulum in* Sapajus*.

## 1. Introduction

**Figure 1:**
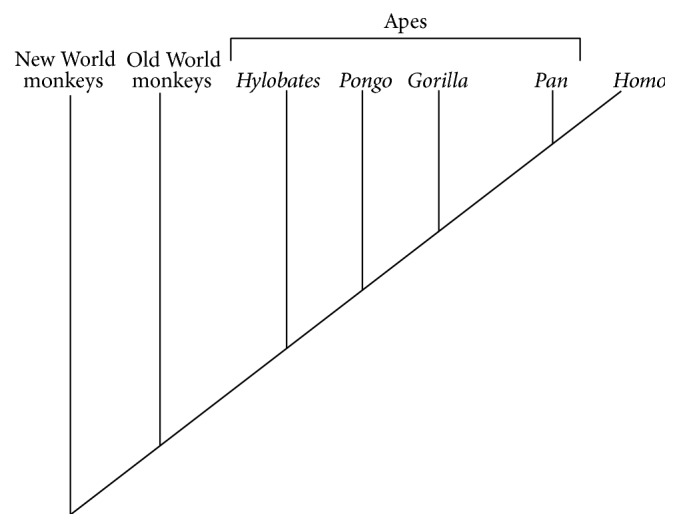
A simple primate cladogram to indicate the distance of the derivation among primates.* Sapajus* is a New World Monkey and* Macaca mulatta* is an Old World Monkey.

The* Sapajus* sp. (bearded capuchins), as an exception among New World primates [1], present high cognition and memory [2], tool use associated with intermittent bipedalism in the captivity and in the wild [3–8], handling rocks to open coconuts [9], and fishing for termites using twigs [10]. Thus, they share a range of behaviors with* Pan* (chimpanzees) and* Homo* (hominids) [11–17] (Figure 1).* Sapajus* also have well-developed brains relative to their body weight [18, 19] and a high degree of motor development [20].

However, recent anatomical studies demonstrated that bearded capuchins do not have true thumb opponency [21], and their anatomical structures are more similar to those of baboons than chimpanzees, except for some features in the forearm muscles [1]. Accordingly, comparison with Old World primates and apes suggests that cognition ability in bearded capuchins is similar to that in chimpanzees [21]. These contradicting findings suggest that more studies are required to explain the unexpected high cognitive abilities in* Sapajus*. Indeed, studies on brain anatomy are scarcer in that genre. The present known evidence in brain anatomy in* Sapajus* does not permit inferring correctly their higher cognitive abilities comparable to other nonhuman primates.

Connectivity between different parts of the brain is one of the important indices for complex brain functions. The brain with complex neural networks can acquire a more elaborate repertoire of behaviors in primates (because of its large size and complexity), resulting in highly sophisticated cultural behaviors in humans, such as language, tool use, and social learning [6, 22, 23]. Thus, primates have well-developed association cortices, and the sensory areas are well separated in the cortex. Many primates have the prefrontal cortex as well as the parietal, temporal, and occipital cortices, all of which have long association fibers that run through the white matter [24–32].

The connections between the frontal and other cortical regions, that is, the association fibers, have been studied in detail in humans and nonhuman primates, using various kinds of techniques [32, 33]. The postmortem blunt fiber dissection is an important initial technique to study association fibers [34]. However, few studies investigated association fibers and white matter in Cebid monkeys, or any other New World primates. In the present study, we investigated association fibers in the brain to characterize anatomical features of this species in comparison with Old World primates,* Macaca mulatta* mainly, and* Homo*. Based on the findings, we discussed evolution of the primate brain and characteristics of* Sapajus* brain among the primates.

## 2. Materials and Methods

### 2.1. Subjects

A total of 24 hemispheres of* Sapajus* (consisting of 12 left antimeres and 12 right antimeres) were used in this study. These specimens were provided by the Department of Surgery, Faculty of Veterinary and Animal Science, University of São Paulo (FMVZ/USP), Brazil. These specimens were derived from wild primates that underwent natural death in the neighborhood of citizens in three different provinces in Brazil. Four adult males and 1 adult monkey of unknown gender (because only the head was received) were obtained in Sete Lagoas, state of Minas Gerais, Southeast of Brazil, in the 1970s decade. Two adult males, 1 young male, and 1 adult female were obtained in the Goiânia, state of Goiás, Midwest of the Brazil (in the proximity of the campus of the Federal University of Goiás) 10 years ago. One adult male, 1 young female and 1 adult female were obtained in Palmas, state of Tocantins, north of Brazil, 3 years ago. Those animals were found by IBAMA (Brazilian Institute of the Natural Resources) and sent to Federal University of Goiás. They had been used in previous studies and they were kept for further use in order to avoid the unnecessary sacrifice of animal lives, in compliance with international standards of bioethics and animal welfare. The research was accomplished in the Federal University of Goias (UFG), Brazil. We declare for any purposes that it may be necessary that the research follows the Principles for the Ethical Treatment of Nonhuman Primates indicated by the guidelines of the American Society of Primatologists (ASP).

### 2.2. Dissection of the Intrahemispheric Fiber Systems of the* Sapajus* Brain

The brains were stored in 10% formaldehyde solution. This fluid was replaced after 24 hours, then the brains were kept in a renewed solution for 30 days. We used Klingler's preservation method with minor adjustments [35, 36]. We also used, as a study reference, the technique by de Castro et al. [37]. The freezing-thawing procedure was repeated three times, which made it easier to prepare for dissections of fiber tracts and nuclei, highlighting the distinction between the gray and white matter. The fiber dissection technique allows three-dimensional understanding of anatomy of the brain. Klingler's method allowed the demonstration of structures that make up the internal anatomy of the fiber systems within the cerebral hemispheres.

According to Klingler's freeze-thaw method, the brains were washed for about 4 hours in water at room temperature. The pia mater, arachnoid, and the vessels of the brains were carefully removed with small tweezers, the brains were immersed in 10% formaldehyde and they were frozen for 8 days at an average temperature of −10°C. The brains were then washed under running water for 24 hours. The freezing procedure (in 10% formaldehyde solution) was repeated three times. After the last freezing process, the brains were kept in 10% formaldehyde solution. The dissections were made using wooden spatulas in different but appropriate sizes and shapes according to the gyri and cerebral sulci dissected (modified from sticks with a length of approximately 25 cm). The spatulas were used for a careful removal of the gray matter. After this procedure, the hemispheres were washed in running water and then they were gently wiped and dried using paper towels. Later, we used pins or sewing needles to follow the path of fibers that were coming from or going towards the prefrontal region.

The characteristics of the fiber systems in each hemisphere were analyzed and photographed both before and after the dissection with a* Canon Power Shot A520*. The photos showed the lateral, medial, and frontal patterns of the anatomic orientation.

## 3. Results

The results showed that there was a large amount of the gray matter in the frontal region as well as in other brain regions. After dissecting the cerebral hemispheres, we found complex patterns of fiber organization beneath the cortex. The technique of fiber dissection proved to be a very useful and safe method.

**Figure 2:**
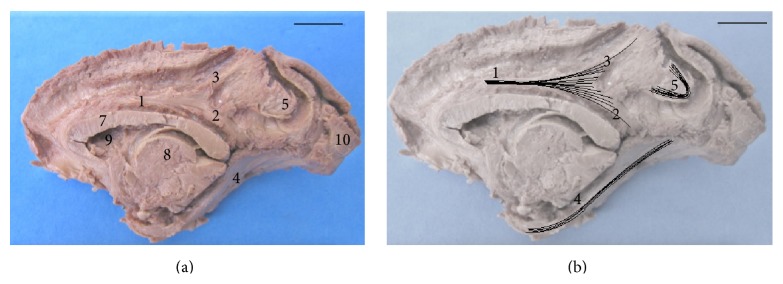
Medial aspect of the right hemisphere. (a) 1: united cingulum and superior longitudinal fasciculus; 2: the cingulum fasciculus; 3: the superior longitudinal fasciculus; 4: the inferior longitudinal fasciculus; 5: the short arcuate fibers (in “U”); 7: the body of corpus callosum; 8: the thalamus; 9: the lateral ventricle; 10: the occipital lobe (bar = 1 cm). (b) Fascicles 1, 2, 3, and 4 are highlighted (bar = 1 cm).

**Figure 3:**
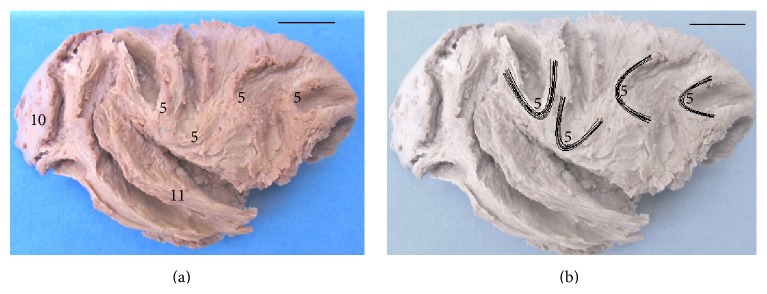
Lateral aspect of the right hemisphere. (a) 5: short arcuate bers (in “U”); 10: occipital lobe; 11: temporal lobe (bar = 1 cm). (b) Fascicles 5 are highlighted (bar = 1 cm).

**Figure 4:**
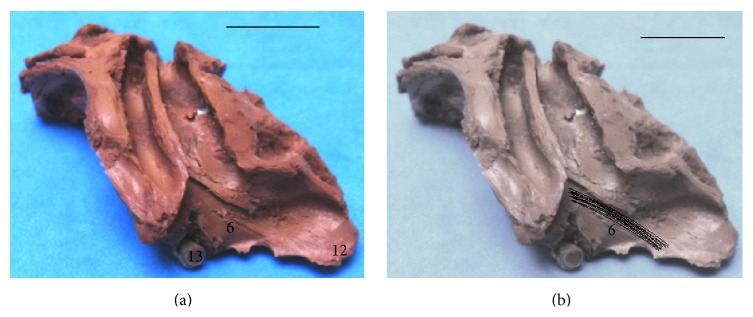
Orbital aspect of the right hemisphere. (a) 6: the uncinate fasciculus; 12: the orbitofrontal region; 13: the optic chiasm (bar = 1 cm). (b) Fascicle 6 is highlighted (bar = 1 cm).

**Figure 5:**
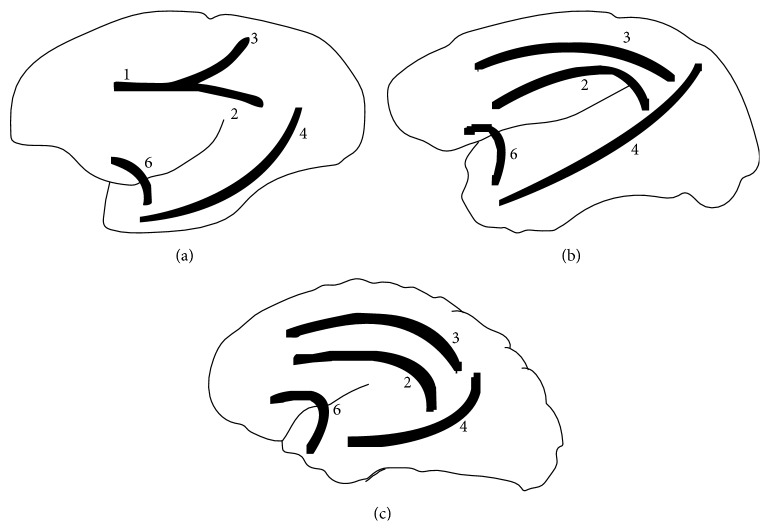
Schematic drawings of the brain of the* Sapajus* (a),* Macaca* (b), and* Homo* (c), indicating the studied fasciculi. 1: united cingulum and superior longitudinal fasciculus; 2: the cingulum fasciculus; 3: the superior longitudinal fasciculus; 4: the inferior longitudinal fasciculus; 6: the uncinate fasciculus.

Dissection of cerebral hemispheres of* Sapajus* showed the same distribution patterns of the fiber systems without anatomical variations. We found a variety of fibers in the white matter, linking the frontal region to several brain regions, mainly the temporal and parietal lobes in the* Sapajus* brain. The cingulum and inferior longitudinal fasciculi as well as the uncinate fasciculus were found in the all cerebral hemispheres. The specimens also showed an arcuate path with fibers around the splenium, body, and genu of the corpus callosum. In a medial view (Figures 2 and 5), we observed short arcuate fibers, the cingulum fasciculus united with the superior longitudinal fasciculus, and inferior longitudinal fasciculus. These structures were seen in all cases in both antimeres. In a lateral view (Figures 3 and 5), we found short arcuate fibers (or “U” fibers) and inferior longitudinal fasciculus. The inferior longitudinal fasciculus displayed a clear pattern of distribution linking the occipital and temporal regions in all the dissected cerebral hemispheres. Short arcuate fibers (in “U” fibers) connecting adjacent gyri were found in the medial view of the occipital lobe and in the lateral view of the frontal and parietal lobes. The uncinate fasciculus that connects the orbital frontal region to the temporal lobe was also observed in an orbital view (Figures 4 and 5).

## 4. Discussion

### 4.1. Anatomical Consideration

The cingulum and superior longitudinal fasciculus were found as the evident major fibers. No previous study investigated the cingulum and intrahemispheric connections in other primates of the New World. The literature on nonhuman primates investigated principally* Macaca mulatta* (rhesus monkeys) [20, 25, 38–44]. In* Macaca* and* Homo*, the cingulum bundle links the cingulate gyrus with the hippocampus and parahippocampal gyrus involved in spatial working memory and motivation and emotional aspects of behaviors [32].

The superior longitudinal fasciculus in* Macaca* and* Homo*, which connects the parietal, occipital, and temporal lobes to the frontal cortex [32, 34], can be divided into 5 subcomponents [45] and is involved in emotions, attention, memory, and language [46]. In the present study, we could not recognize such 5 subcomponents of the superior longitudinal fasciculus at least by visual inspection. Furthermore, the superior longitudinal fasciculus seems to be reduced in* Sapajus* compared with that in* Homo*. Since the superior longitudinal fasciculus is important as a “language pathway” [34], this might be attributed to highly primitive ability of language in these monkeys, which requires less associative connections.

The cingulum had upward traffic directed to the posterior region of the brain observed in the medial view in* Sapajus*, which is similar to the traffic in* Macaca* and* Homo* [20, 25]. However, the cingulum united with the superior longitudinal fasciculus in its anterior-ventral part in* Sapajus*, which is unprecedented in other primates as far as we know. Although the superior longitudinal fasciculus and cingulum are separated in* Homo* in a lateral view [20], such two separated fiber systems were not observed in* Sapajus*. It is important to note that the cingulum bundle and the superior longitudinal fasciculus share the same associative functions such as memory and emotions in* Macaca* and* Homo* [32], suggesting that both fasciculi might take similar trajectory in an initial stage of brain development. However, no lateral expansion of the brain happened in* Sapajus* in contrast to* Homo*, because of their evolutionary option, which might result in unification of cingulum and the superior longitudinal fasciculus.

The inferior longitudinal fasciculus observed in the present study was also reported in* Macaca* and* Homo*, where its functions are related to recognition and discrimination of faces and objects and its memory [32]. The uncinate fasciculus connecting the frontal and temporal lobes observed in the present study was also reported in* Macaca* as well as* Homo* [32]. This fasciculus is suggested to be involved in processing new information, understanding emotional aspects of the sounds, regulation of emotions, and interaction between emotion and cognition [32]. Although we observed short arcuate fibers (in “U” fibers) connecting adjacent gyri, these fiber bundles were less frequent in* Sapajus* than in* Homo*. This might be ascribed to the anatomical differences between the two species where the* Homo* brain had much richer cortical circumvolutions than* Sapajus* [18].

### 4.2. Evolutionary Consideration

The present study reported similar size and amount of association fibers originating from the frontal lobe in* Sapajus* to those in* Macaca* and* Homo*. The frontal cortex in* Sapajus* keeps high percentage in its brain [47]. Extensive studies reported that* Sapajus* monkeys have similar relative neocortical size as in big apes and are highly encephalized [8, 15, 20, 48–50].

Schoenemann et al. [50] compared brains of several primate species including* Sapajus* with those in* Homo sapiens* as a control group. They reported that the percentage of the white matter in* Homo sapiens* was significantly larger than all the other primate species, except for* Gorilla gorilla* (gorilla) and* Sapajus apella*, although the gray matter did not show significant differences. This suggests that the complexity of fiber connections with other cortical areas played a key role in the evolutionary process of the primate brain and behaviors. In the present study, the cingulum bundle united with the superior longitudinal fasciculus in* Sapajus*. This might suggest that brains of the New World primates are more primitive than* Macaca* and* Homo* primates compared here, although no other data are presently available. In fact, the results in this paper are insufficient to support the high cognition observed in* Sapajus*, mainly because few data for comparison are available in other primates. Indeed, this work is the first to investigate association fibers in New World primates, and further studies are required to understand the nature and evolution of the white matter in primates.
